# The spoke wheel color Doppler blood flow signal is a specific sign of papillary thyroid carcinoma

**DOI:** 10.3389/fendo.2022.1030143

**Published:** 2022-10-25

**Authors:** Nianyu Xue, Ping Li, Huadong Deng, Jing Yi, Yu Xie, Shengmin Zhang

**Affiliations:** ^1^ Department of Ultrasonography, Ningbo First Hospital, Ningbo, Zhejiang, China; ^2^ Department of Ultrasonography, Nanjing First Hospital, Jiangsu, China; ^3^ Department of Ultrasonography, Lishui People's Hospital, Zhejiang, China; ^4^ Department of Ultrasonography, Renmin Hospital of Wuhan University, Wuhan, Hubei Province, China; ^5^ Department of Ultrasonography, Meishan People’s Hospital, Meishan, China

**Keywords:** thyroid neoplasms, ultrasonography, doppler, color, papillary thyroid carcinoma

## Abstract

**Background:**

Papillary thyroid carcinoma (PTC) is the most common type of thyroid cancer. Grayscale ultrasound (US) is the main method used to diagnose benign and malignant thyroid nodules, While color doppler blood flow imaging(CDFI) is not widely recognized when diagnosing thyroid cancer.

**Methods:**

This study used a retrospective analysis. The study included 36 spoked wheel blood flow nodules detected by CDFI in 37,372 patients in five hospitals from January 2020 to June 2021. All thyroid nodules were examined histologically after ultrasound-guided fine needle biopsy or following surgical resection. The value of color doppler in diagnosing papillary thyroid carcinoma was evaluated based on pathological results.

**Results:**

Among 36 thyroid nodules, only 6 were highly suspected of being malignant on grayscale ultrasound (classified as 5, according to ACR TI-RADS). However, these 36 thyroid nodules showed spoke wheel blood flow signal distribution on CDFI. If the spoke wheel blood flow signal is used to diagnose papillary thyroid cancer, then the diagnostic accuracy of this group of papillary thyroid cancers can reach 100%, which is significantly higher than the accuracy of grayscale ultrasound diagnosis, and the difference is statistically significant (p<0.05).

**Conclusions:**

The results of this study found that spoke wheel blood flow sign on CDFI can be used to diagnose PTC. PTC with spoke wheel blood flow have benign characteristics on gray-scale ultrasound, which is easy to be misdiagnosed.

## Introduction

Thyroid nodules are considered to be the most common manifestation of endocrine disease in clinical practice. The incidence of thyroid cancer has rapidly increased throughout the past few decades (1). The increase in the detection of malignant thyroid tumors may be related to the widespread use of high-frequency ultrasound, which is the most preferred diagnostic method for thyroid nodules (2).

Color Doppler blood flow imaging (CDFI) is an imaging technique that exploits the shift in the frequency of US waves when waves are reflected by moving blood (the Doppler effect) (3). In CDFI, the Doppler effect can detect the movement of red blood cells in blood vessels. Therefore, CDFI can provide information on the vascularity of thyroid lesions and is widely used in daily clinical practice to evaluate diffuse thyroid diseases, such as hyperthyroidism. However, CDFI is not valuable in assessing benign and malignant thyroid nodules (4). CDFI information is not considered in the standards of various versions of thyroid classification (including ACR, Korean quark classification, Chinese version classification, etc.) (5–8), which also proves that CDFI is of no value in the differentiating benign and malignant thyroid nodules. Ultrasonographic findings related to malignant tumors included hypoechoicity, solid and irregular edges, taller-than-wide and calcification.

In reviewing cases of misdiagnosed thyroid tumors, the authors found that papillary thyroid carcinomas with spoke wheel blood flow were easily misdiagnosed as benign nodules. To the best of our knowledge, there are no reports in the literature regarding the use of CDFI spoke wheel blood flow distribution to distinguish benign and malignant thyroid nodules. Therefore, we conducted a study that evaluated the value of spoke wheel blood flow distribution in the diagnosis of papillary thyroid carcinoma.

## Material and methods

### Study population

The study was approved by the ethics committee of our hospital(NO.2022RS042). Individual consent for this retrospective analysis was waived.

From January 2020 to June 2021, among 37,372 thyroid nodules in five hospitals, 38 nodules with spoke wheel blood flow detected by color Doppler ultrasonography were included in the study. The selection criteria were as follows: all patients underwent grayscale ultrasound and color Doppler ultrasound examinations, and pathological results were obtained. Among the 38 patients, 2 patients with thyroid nodules did not obtain pathological results, and the remaining 36 patients (20 males and 16 females, age range, 17–65 years; mean age, 36.5 ± 12.2 years) were included in this study. The patients with thyroid nodules with spoke wheel blood flow were all single. The mean diameter of the thyroid nodules was 2.1 ± 1.1 cm (range, 0.6–4.6 cm). All thyroid nodules were examined histologically after ultrasound-guided fine needle biopsy or following surgical resection.

### Ultrasound examinations

All patients in the study underwent grayscale ultrasound and color Doppler ultrasound. When a thyroid nodule was detected, the position, size, echogenicity, composition, margin, shape, echogenic foci and blood flow of the nodule (spoke wheel blood flow distribution and non-spoke wheel blood flow distribution) were recorded. According to the American College of Radiology (ACR) Thyroid Imaging Reporting and Data System (TI-RADS) classification. An ultrasound machine (including Toshiba, GE, Mindray, Philips, Hitachi) with high frequency (7-14 MHz) line array transducers was used in all imaging procedures.

Ultrasound imaging was performed by the same radiologist, who had more than five years of experience in thyroid ultrasound, in each hospital. All images were recorded and transferred to the research staff database. Then, the images were evaluated by two radiologists who had 10 years of experience in thyroid imaging. If there was any disagreement, a third senior radiologist, who had more than 15 years of experience in thyroid ultrasonography, was consulted until a consensus was reached. None of the three radiologists knew the pathological diagnosis of the thyroid nodules.

**Figure 1:**
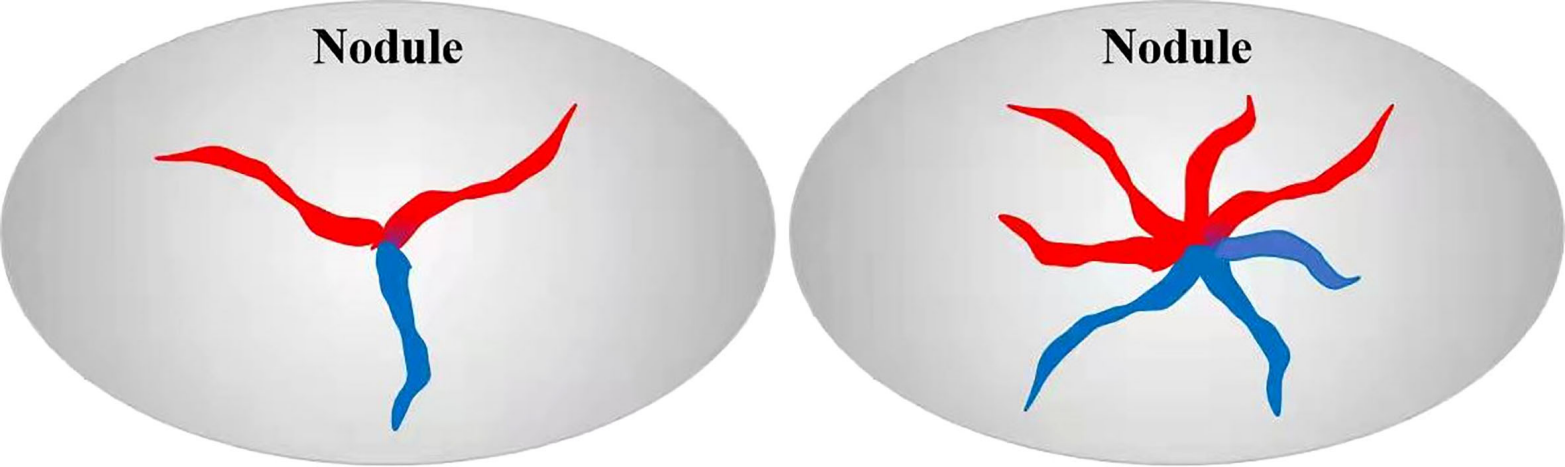
Schematic diagram of spoke wheel blood flow. Red: blood flow towards the probe, blue: blood flow away from the probe.

First, thyroid nodules were diagnosed based on grayscale ultrasound findings, and then thyroid papillary carcinoma was diagnosed based on the color Doppler spoke wheel blood flow distribution. The value of color Doppler in diagnosing papillary thyroid carcinoma was evaluated based on pathological results. Color Doppler spoke wheel blood flow distribution was determined based on the observance of at least three blood vessels in different directions from the center of the nodule to the periphery (**Figure 1**).

### Pathological examination

After the ultrasound examination was completed in all the patients, ultrasound-guided fine-needle aspiration or surgical resection was performed within two weeks to obtain pathological results.

### Statistical analysis

The chi-squared test or Fisher’s exact test was applied to categorical variables. The statistical analysis was performed, and histopathology results were considered the diagnostic gold standard. *P value*s *<*0.05 were considered to be statistically significant. Data analysis was performed using the SPSS version 20.0 software package.

## Results

A total of 37,372 thyroid nodules were examined, of which 38 with spoke wheel blood flow signals were detected. Two of the patients were refused further examination, thus, no pathological results were obtained. The remaining 36 patients underwent fine-needle aspiration biopsy or surgical resection, and the pathological results confirmed that each patient had papillary carcinoma. Although the incidence of spoke wheel blood flow signals in thyroid nodules is very low (0.1%), it can directly diagnose papillary thyroid carcinoma (specificity as high as 100.0%).

**Figure 2:**
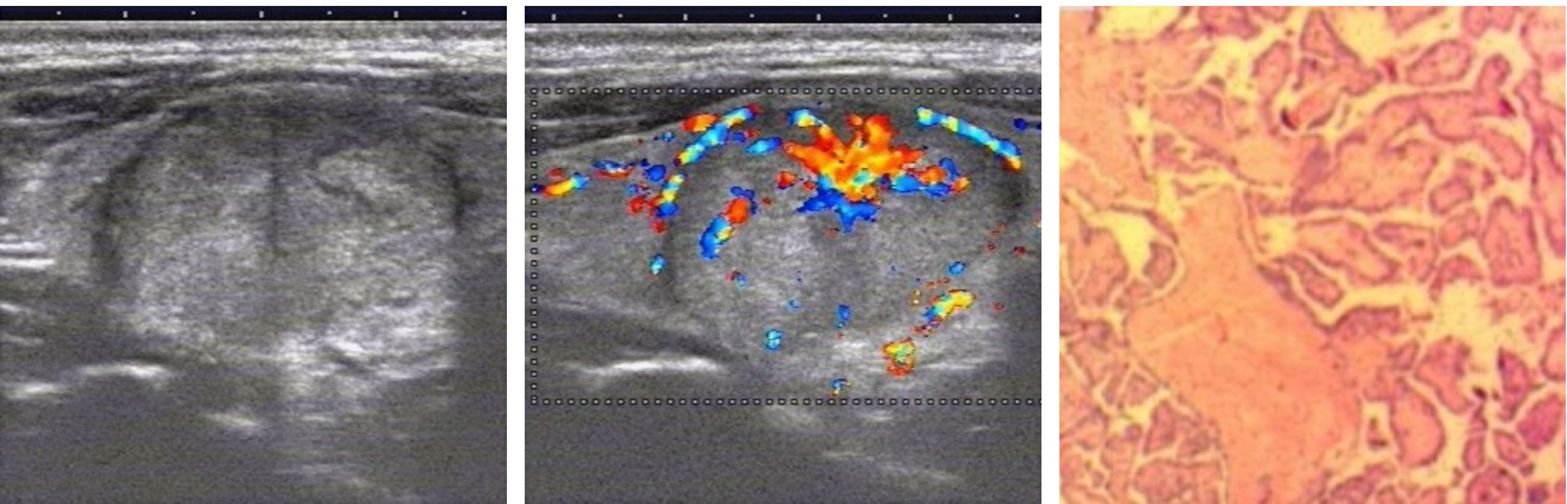
Grayscale ultrasonography shows a solid, isoechoic nodule in the right lobe of the thyroid with wider-than-tall and smooth margin and is considered to be a benign nodule on grayscale ultrasonography. Color Doppler ultrasonography showed the distribution of spoke wheel blood flow signals, which was determined to be papillary thyroid carcinoma. Postoperative pathology confirmed papillary thyroid carcinoma.

**Figure 3:**
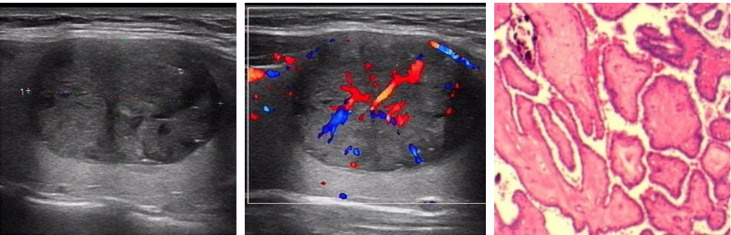
Grayscale ultrasonography shows a solid, hypoechoic nodule in the right lobe of the thyroid with wider-than-tall and smooth margin. It was classified as an ACR TI-RADS 4 lesion with moderate suspicion of cancer requiring biopsy. Color Doppler ultrasonography showed the distribution of spoke wheel blood flow signals, which was determined to be papillary thyroid carcinoma. Postoperative pathology confirmed papillary thyroid carcinoma.

We analyzed and summarized the ultrasound imaging characteristics of these 36 patients with papillary thyroid carcinoma (**Table 1**). Thirty-six thyroid nodules were identified and no cervical lymph node metastasis was found before ultrasonography. Among the 36 thyroid nodules, only 6 were highly suspected of being malignant on grayscale ultrasound (classified as 5, according to ACR TI-RADS), 15 nodules were identified as indeterminate benign and malignant (classified as 4, according to ACR TI-RADS), and the remaining 15 were determined to be benign (classified as Class 3, according to ACR TI-RADS). However, these 36 thyroid nodules showed spoke wheel blood flow signal distribution on color Doppler blood flow imaging (**Figures 2**, **3**). The spoke wheel blood flow signal was not present in benign thyroid nodules. Therefore, the spoke wheel blood flow signal can be used as a specific ultrasound manifestation of papillary thyroid carcinoma.

**Table 1:** Ultrasonic image features of 36 cases of papillary thyroid carcinoma with spoke wheel blood flow.

Characteristics		Number
Gender	Female Male	16 20
Age (year)		36.50 ± 12.15
Location	Right lobe Left lobe	21 15
Maximum diameter of lesion (cm)		2.05 ± 1.09
Echogenicity	Hypoechoic Isoechoic	13 23
Composition	Mixed cystic and solid Solid or almost completely solid	3 33
Margin	Smooth Lobulated or irregular	33 3
Shape	Taller-than-wide Wider-than-all	0 36
Echogenic foci	Punctate echogenic foci Macrocalcifications None	10 5 21
Color doppler flow imaging	Spoke wheel blood flow	36

If the spoke wheel blood flow signal is used to diagnose patients with papillary thyroid cancer, the diagnostic accuracy of this group of papillary thyroid cancers can reach 100.0%. This was significantly higher than the accuracy of grayscale ultrasound diagnosis (16.7%), and the difference was statistically significant (*p*<0.05). Regardless of the gray-scale ultrasonic characteristics, as long as the thyroid nodule has a spoke like blood flow, it can be divided into ACR TI-RADS 5.

## Discussion

Papillary thyroid carcinoma (PTC) is the most common type of thyroid cancer (9, 10). Grayscale ultrasound (US) is the main method for diagnosing benign and malignant thyroid nodules. The main ultrasound features that suggest PTC are hypoechoic or very hypoechoic, solid component, lobulated or irregular margins or extra-thyroidal extension, taller-than-wide shape, and calcifications. As greater numbers of the above ultrasound features are observed, the probability of malignancy increases. These ultrasound features are used for risk classification of thyroid imaging reports and data systems. Clearly, color Doppler blood flow imaging is not recognized in various versions of the thyroid risk classification. This suggests that color Doppler blood flow information is not used for the diagnosis of thyroid cancer. Both benign and malignant thyroid nodules can present with or without abundant blood flow.

This study found that the CDFI spoke wheel blood flow signal distribution can be used as a highly specific sign of papillary thyroid carcinoma. Retrospective analysis of the characteristics of this type of papillary thyroid carcinoma was performed. Incidence rates were not significantly different regarding sex and location. Similar to the reported papillary thyroid gland in the literature, the age of onset is approximately 36 years (11–14). The maximum diameter of the mass in this group of patients was approximately 2 cm, which is larger than that reported in the literature on papillary thyroid carcinoma (12–14). This group of papillary carcinomas are speculated to have a rich blood supply and are easier to grow up. However, we did not follow up on its growth rate. The grayscale ultrasound features of this group of PTCs are mainly characterized by nodules that are isoechoic, solid, smooth margin, with a wider than tall, no calcifications and no halo. Except for the solid component, there are few other malignant indicators (hypoechoic or very hypoechoic, solid component, lobulated or irregular margins or extra-thyroidal extension, taller-than-wide shape, and calcifications), so grayscale ultrasound findings can easily suggest that the nodules are benign. In this study, the misdiagnosis rate of gray-scale ultrasound in 36 cases of papillary thyroid carcinoma was significantly higher than that of papillary thyroid carcinoma reported in the literature (15). If the spoke wheel blood flow signal is used to diagnose papillary thyroid cancer, the diagnostic accuracy of this group of papillary thyroid cancers can reach 100%. CDFI can significantly improve the diagnostic accuracy of this type of PTC. The spoke wheel blood flow mainly appears in the larger PTC. PTC papillary structure has fiber vascular axis. Large fiber vessels will branch out. If the branch vessels originate from the center of the mass and the vessels are large enough, they can be detected by CDFI.

This study had several limitations. First, the case samples were limited, and large samples are needed for further research and confirmation. Second, we did not follow up patients for a long time to observe their outcomes. Third, limited by the retrospective study, there is a unified standard for image acquisition of spoke wheel blood flow, which may affect its incidence.

## Conclusions

The results of this study found that CDFI can be used to diagnose papillary thyroid cancer that have characteristics of spoke wheel blood flow distribution and using grayscale ultrasound for these cases of PTC often show benign features and is easily misdiagnosed.

## Data availability statement

The raw data supporting the conclusions of this article will be made available by the authors, without undue reservation.

## Ethics statement

The studies involving human participants were reviewed and approved by Ethics Committee of Ningbo First Hospital. Written informed consent for participation was not required for this study in accordance with the national legislation and the institutional requirements.

## Author contributions

SZ and NX designed this study. NX, PL, HD, JY, YX and SZ conducted the study and collected important background data. NX and SZ drafted the manuscript. All authors contributed to the article and approved the submitted version.

## Conflict of interest

The authors declare that the research was conducted in the absence of any commercial or financial relationships that could be construed as a potential conflict of interest.

## Publisher’s note

All claims expressed in this article are solely those of the authors and do not necessarily represent those of their affiliated organizations, or those of the publisher, the editors and the reviewers. Any product that may be evaluated in this article, or claim that may be made by its manufacturer, is not guaranteed or endorsed by the publisher.
